# Molecular survey of vector-borne diseases in two groups of domestic dogs from Lisbon, Portugal

**DOI:** 10.1186/s13071-021-04650-4

**Published:** 2021-03-18

**Authors:** Ana Mafalda Dordio, Relja Beck, Telmo Nunes, Isabel Pereira da Fonseca, Jacinto Gomes

**Affiliations:** 1 CIISA–Centro de Investigação Interdisciplinar em Sanidade Animal, Universidade de Lisboa, Lisbon, Portugal; 2 Croatia Veterinary Institute, Zagreb, Croatia; 3 Instituto Nacional de Investigação Agrária e Veterinária, Oeiras, Portugal

**Keywords:** Canine vector-borne diseases (CVBD), Dogs, Molecular methods, Southern Portugal

## Abstract

**Background:**

Canine vector-borne diseases (CVBDs) are caused by a wide range of pathogens transmitted by arthropods. They have been an issue of growing importance in recent years; however, there is limited information about the vector-borne pathogens circulating in Portugal. The aim of the present study was to detect canine vector-borne bacteria and protozoa of veterinary and zoonotic importance using molecular methods.

**Methods:**

One hundred and forty-two dogs from Lisbon, southern Portugal, were tested: 48 dogs from a veterinary hospital clinically suspected of vector-borne diseases and 94 apparently healthy dogs from shelters. *Anaplasma* spp./*Ehrlichia* spp., *Babesia/Theileria* spp., *Hepatozoon* spp., and *Mycoplasma* spp. infections were detected by PCR from blood samples and examined under light microscopy. Other information including clinical status and diagnostic test results were collected for each animal.

**Results:**

Infections were detected by PCR in 48 (33.80%) dogs. Single infections were found in 35 dogs (24.64%), and co-infections were found in 13 (9.15%) dogs. Twenty-nine (20.42%) dogs were positive for *Hepatozoon* spp., 15 (10.56%) for *Mycoplasma* spp., 11 (7.75%) for *Anaplasma* spp./*Ehrlichia* spp., and six (4.21%) for *Babesia* spp. DNA sequencing was used to identify *Babesia vogeli* (2.81%), *Babesia canis* (1.40%), *Hepatozoon canis* (20.42%), *Mycoplasma haematoparvum* (2.11%), *Mycoplasma haemocanis* (8.45%), *Anaplasma platys* (7.04%), and *Ehrlichia canis* (0.70%).

**Conclusions:**

This is the first molecular identification of *B. canis* and *M. haematoparvum* in dogs from southern Portugal. This study highlights the importance of molecular methods to identify CVBD pathogens in endemic areas and helps to guide the clinical approach of veterinarians in practice.

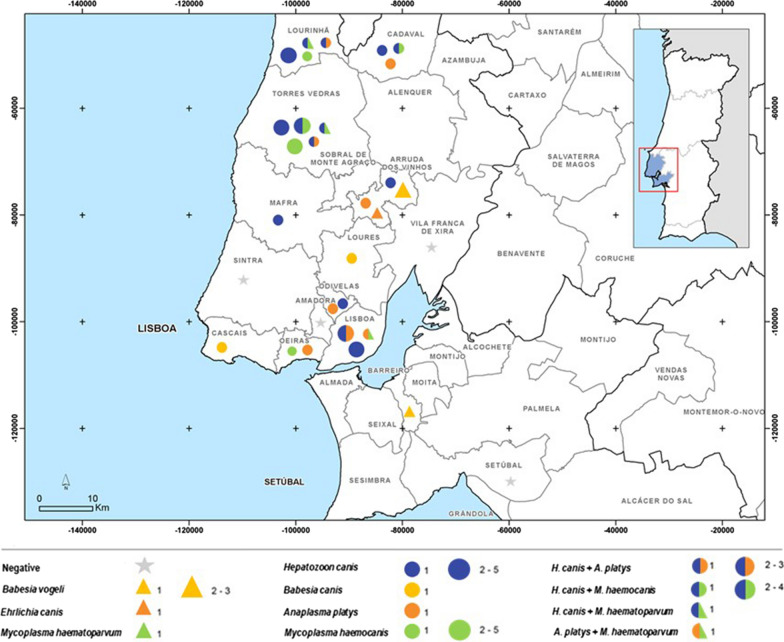

## Background

Canine vector-borne diseases (CVBDs) are caused by a wide range of pathogens, including viruses, bacteria, protozoa, and helminths. These are transmitted to dogs by different species of arthropod vectors. Prevalence of vector-borne diseases (VBDs) may increase in certain areas, either due to the importation of infected animals or due to spread and establishment of the causative pathogens and their vectors in previously non-endemic areas. CVBD pathogens constitute a diagnostic challenge for practitioners due to limitations of overlapping of clinical signs, detection limits of diagnostic methods, co-infections with more than one pathogen, and the emergence of new pathogens [1, 2]. In dogs, different co-infections occur frequently in endemic areas and may partially explain variations in clinical presentation, pathogenicity, and response to therapy [3].

To date, five species of *Babesia* have been identified by molecular methods in dogs in Europe: *Babesia canis*, *Babesia vogeli*, *Babesia gibsoni*, “*Babesia vulpes”*, and *Babesia caballi* [4, 5]. In southern Europe, *B. vogeli* is the most commonly detected species transmitted by the brown dog tick, *Rhipicephalus sanguineus* [6], while *B. canis* is associated with *Dermacentor reticulatus* [7]. *Ixodes hexagonus* and *Ixodes canisuga* have been proposed as vectors of *“B. vulpes”* [8, 9]. *Babesia canis*, *Babesia* vogeli, and “*Babesia vulpes”* DNA were found in dogs from northern Portugal [10, 11], whereas in southern Portugal, only DNA from *B. vogeli* was reported [12]. Seroprevalence of *B. canis* reported from autochthonous Portuguese dogs was 58% [13].

Morphologically, piroplasms are divided into two groups based on merozoites size: small forms (1.0–2.5 µm) that include *B. gibsoni* and “*B. vulpes*” and large piroplasms (2.5–5.0 µm) including *B. rossi*, *B. canis*, and *B. vogeli*. The clinical signs and prognosis of babesiosis vary depending on the specie causing infection. Disease severity is moderate to severe for infections caused by *B. canis*, *B. gibsoni*, and “*B. vulpes*”. While the severity for *B. vogeli* infections is mild to moderate and with good prognosis. Other factors that influence prognosis are age, immune competence, presence of concomitant infection or disease, and whether or not the animal has undergone splenectomy [14, 15]. The common findings associated with *Babesia* species are anorexia, lethargy, fever, lymphadenopathy, splenomegaly, anaemia, icterus, thrombocytopenia, and haematuria. The molecular characterisation of species is also important for medical treatment since they have different susceptibility to anti-protozoal drugs [15].

Hepatozoonosis caused by *Hepatozoon canis* is another CVBD, reported in the Old World and more recently from South and North America [16]. *Hepatozoon canis* has already been molecularly detected in dogs from the north [10] and has been highly prevalent in the south of Portugal [12]. The transmission of this apicomplexan parasite to the vertebrate hosts typically takes place by ingestion of the arthropod vector *R. sanguineus* containing mature protozoal oocysts with infective sporozoites [17]. In dogs, infection with *H. canis* is often subclinical, but may manifest as a mild to debilitating and even life-threatening disease with cachexia, lethargy, and anaemia [1]. In cases with high parasitaemia levels, the animals present changes in the complete blood count (CBC), such as leucocytosis, neutrophilia, and anaemia. *Hepatozoon canis* can be detectable by microscopic observation of circulating intracellular gamonts in stained blood smears [17].

Haemotropic mycoplasmas are small, uncultivable, cell wall-less bacteria, and there are two species most described in molecular studies from dogs: *Mycoplasma haemocanis* and *Mycoplasma haematoparvum*. Although their transmission routes are poorly understood, *R. sanguineus* has been suggested as a potential tick vector [18]. *Mycoplasma haemocanis* has been reported in Portugal with a prevalence of 40% [18]. Most infected dogs present with chronic and asymptomatic infections, but acute infection can lead to haemolytic anaemia with varying levels of severity [18].

There are other bacteria associated with CVBDs within the Anaplasmataceae family, including *Ehrlichia*, *Anaplasma*, *Neorickettsia*, and *Wolbachia* [19]. Canine monocytic ehrlichiosis, caused by the bacterium *Ehrlichia canis*, is one of the CVBDs with the most severe clinical signs in dogs, and considered endemic in European countries bordering the Mediterranean Sea. *Ehrlichia canis* transmission is primarily associated with *R. sanguineus* [20]. However, in Portugal, it has also been detected in *Hyalomma* and *Ixodes* species (Pereira da Fonseca, personal communication). *Ehrlichia canis* has been molecularly detected in dogs from both the north [10] and the south of Portugal [12]. Seroprevalence at the national level ranged from 4.1% in apparently healthy dogs to 16.4% in animals clinically suspected of a CVBD [21]. Common clinical signs of ehrlichiosis include anaemia, epistaxis, petechiae, ecchymoses, haematuria, or melena associated with thrombocytopenia, thrombocytopathy, or vasculitis. Detecting *E. canis* morula on blood smears is challenging since it occurs in about 4–6% of clinical cases. For this reason, further diagnostic tests must be conducted, such as serology or molecular techniques (PCR) [22].

In Europe, currently, two species of *Anaplasma* have been reported: *Anaplasma phagocytophilum* and *Anaplasma platys* [22]. *Anaplasma phagocytophilum* has been detected using molecular methods in dogs from the north of Portugal [23] and detected in ticks, *Ixodes ventalloi*, from the south of Portugal [24]. This pathogen can be transmitted by *Ixodes ricinus* and is questionable if *I. ventalloi* is involved in the life cycle [23]. *Anaplasma phagocytophilum* is responsible for canine granulocytic anaplasmosis [21]. In southern Europe, infections caused by *A. platys* are more common, possibly because the presumed vector *R. sanguineus* is widely distributed, and the information regarding prevalence is limited based on a molecular analysis [21]. *Anaplasma platys* has been detected by molecular methods in dogs living in the north and south of Portugal [12, 25]. *Anaplasma platys* is responsible for canine thrombocytotrophic anaplasmosis disease. After 1–2 weeks of infection with *A. platys*, signs of thrombocytopenia and fever can occur. The detection of a morula in platelets or megakaryocytes is possible after 8–15 days of infection. This, however, has a low sensitivity due to the cyclic character of thrombocytopenia, the low percentage of infected cells, and the dependence on examiner experience [26, 27]. National seroprevalence of *Anaplasma* spp. has ranged from 4.5% in apparently healthy dogs to 9.2% in dogs presenting clinical signs [21].

*Ehrlichia canis* and *Anaplasma phagocytophilum* belong to the list of CVBDs with major zoonotic concern, which constitute an emerging worldwide public health threat for pet dogs and their owners [1].

Taking all of this into consideration, a comprehensive molecular survey was conducted to assess the presence of the Anaplasmataceae family, *Babesia* spp., *Hepatozoon* spp., and *Mycoplasma* spp. from apparently healthy and clinically unwell dogs from southern Portugal.

## Methods

### Geographic characterisation and animals selected

From September 2016 to July 2017, blood samples were collected from 142 dogs from Lisbon, southern Portugal, characterised by a temperate climate with both urban and rural settings. The Lisbon district has an area of 2.761 km_2_ divided into 16 municipalities, with a population of two million inhabitants, according to Statistics Portugal (INE).

All dog owners gave written consent after being informed about the objectives of the study. The present study followed the Council of the European Union Directive 86/609/EEC and was approved by the Ethics and Animal Welfare Committee of the Faculty of Veterinary Medicine, University of Lisbon.

Samples were obtained from 48 domestic dogs (group 1) attending the Teaching Hospital of the Faculty of Veterinary Medicine of the University of Lisbon and from 94 apparently healthy dogs from animal shelters (group 2).

The animals of group 1 were included according to the following criteria: suspicion of CVBD with two suggestive clinical signs such as pale mucous membranes, lethargy, anorexia, fever, icterus, and pigmenturia. Since they are non-specific, at least two of these clinical signs were required for inclusion in the study.

For each dog selected, veterinarians had to sample blood for diagnostic confirmation and to fill a registration form gathering anamnesis, description of clinical signs, results of additional tests such as serology, and additional epidemiological information such as the travel history. Physical examination was performed prior to blood collection. Serology results were collected for discussion in the present study, and the samples were considered positive for *Babesia* spp. in serum dilution of 1:32 in indirect immunofluorescence test.

A second group was chosen consisting of apparently healthy dogs from shelters, run by local authorities or animal protection associations in different municipalities of the Lisbon district: Lisbon, Amadora, Odivelas, Arruda dos Vinhos, Torres Vedras, Lourinhã, and Cadaval. Thus giving better insight on species diversity infecting dogs, since analysing dogs with symptoms could bias the survey towards more pathogenic species [4]. In this group, only dogs with previously detected ticks were included. Data of each animal about region, breed, gender, age, living conditions, tick control, and clinical history was registered.

Whole blood samples (1–2 ml) were collected by cephalic or jugular venipuncture into tubes coated with EDTA. Samples were kept initially at 4 °C and later stored at −20 °C until molecular processing in the laboratory.

### Direct examination under light microscopy

EDTA blood was used to prepare thin glass-slide smears that were air-dried, fixed with methanol, Giemsa-stained, and then examined under light microscopy (magnification of 1000×). Blood collected from group 1 was analysed by complete blood count (CBC), while only microhaematocrit plus total protein analysis was performed on blood from group 2. Dogs from both groups were classified as anaemic (PCV < 37%) or non-anaemic (PCV > 37%). The anaemic group was further classified into severely anaemic (PCV < 18%), moderately anaemic (PCV 18–29%), or mildly anaemic (PCV 30–36%).

### Molecular analysis

Using the DNA ‘Blood and tissue kit’ (Qiagen, Hilden, Germany), 200 µl of DNA was extracted from the blood samples from each dog. Further DNA extraction followed the kit manufacturer’s instructions, and the DNA yield was as expected. The presence of DNA from *Anaplasma* spp./*Ehrlichia* spp., *Babesia* spp., *Hepatozoon* spp., and *Mycoplasma* spp. was tested by conventional PCR, with the primers described in Table 1. The same primer sets were then used for sequencing. PCR reaction mixtures of 20 µl were prepared containing 10 µl G2 GOTaq master mix (Promega, Madison, WI, USA), 7.2 µl of DNase/RNase-Free distilled water (Qiagen, Hilden, Germany), 0.4 µl of 10 pmol/µl of each primer, and 2 µl of DNA sample. The successful amplification of the PCR product was confirmed by capillary electrophoresis (QIAxcel; Qiagen, Hilden, Germany) using a QIAxcel DNA Fast Analysis kit, alignment markers (DNA QXAlignmentMarker15bp/3 kb), and QX DNA Size Marker 50–3000 bp. Amplified PCR products were purified using EXOSAP-it^®^ (USB^®^ Products Affymetrix, Inc., OH, USA) according to the manufacturer’s instructions and sequenced in both directions (Macrogen, Amsterdam, The Netherlands). The resulting sequences were assembled using the SeqMan Pro software, edited with Edit Seq tools in Lasergene (DNASTAR, Madison, WI, USA) and compared with available sequences using BLAST in GenBank.

**Table 1:** Primers sets for DNA amplification and sequencing of CVBD pathogens in dogs

Gene target	Pathogen	Sequence (5′-3′)	Fragment length (bp)	Reference
16S rRNA	Anaplasmataceae	EHR16SD: GGT ACC YAC AGA AGA AGT CC EHR16SR: TAG CAC TCA TCG TTT ACA GC	345	Parola et al. [49]
18S rRNA	*Babesia* sp.	BAB F: GTCTTGTAATTGGAATGATGG BAB R: CCAAAGACTTTGATTTCTCTC	550	Beck et al. [4]
18S rDNA	*Hepatozoon* sp.	Hep F: ATACATGAGCAAAATCTCAAC Hep R: CTTATTATTCCATGCTGCAG	666	Inokuma et al. [50]
16S rRNA	*Mycoplasma* sp.	Myco322s: GCCCATATTCCTACGGGAAGCAGCAGT Myco938as: CTCCACCACTTGTTCAGGTCCCCGTC	560	Varanat et al. [51]

### Statistical analysis

Sample size to estimate a simple proportion, apparent prevalence in the apparently healthy population, was calculated for an expected prevalence (*P*) of 6%, an absolute precision of 0.05% (e), and a 95% confidence interval (CI) using the formula *n* = [Z2 × *P* × (1 − *P*)]/e2. The sample size for the clinically suspected population was dependent on the number of dogs that met the criteria during the study period. To compare two sample proportions, the 2-sample z test was used. A *P* value < 0.05 was regarded as statistically significant. The prevalence analysis was performed using CIs, with a 95% CI, using the Wilson method as a CI method. Measuring agreement between molecular methods and blood smear examination was calculated by Cohen’s kappa (κ) and analysed with Landis & Koch, 1977 criteria.

Although the study included two groups, the prevalence was calculated only for the apparently healthy dog group. In this study, we consider a positive result in dogs with a positive conventional PCR result.

## Results

**Fig. 1:**
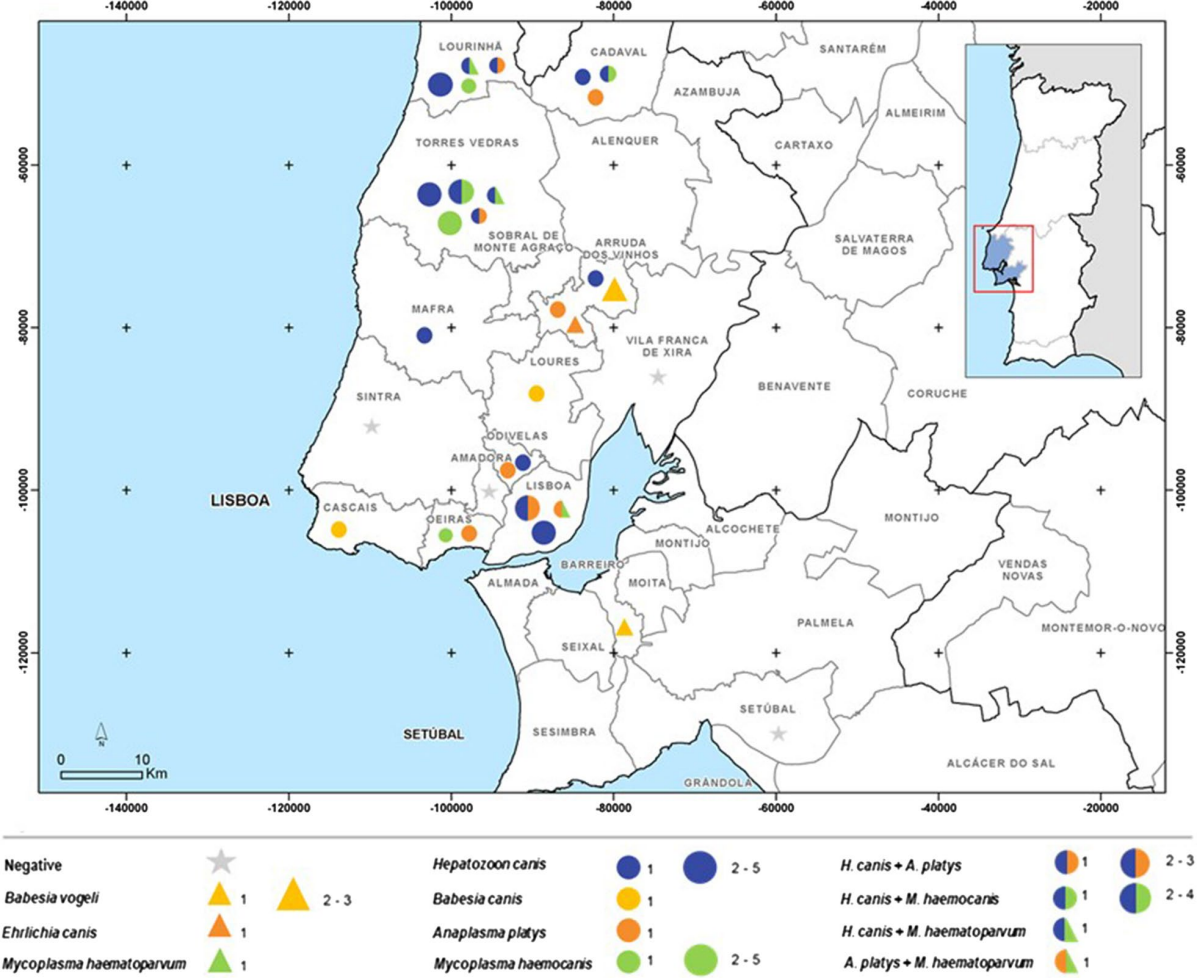
Geographical distribution of CVBD pathogens detected in blood samples by PCR in different counties in the Lisbon district (original Dordio, 2017)

The results, summarised in Table 2 and Fig. 1, revealed infected dogs in both groups. Infections were detected by PCR in 48 (33.80%; CI 26.5–41.9%) dogs. Infections with a single pathogen were found in 35 (24.6%; CI 19.3–32.3%) out of 142 dogs tested: *Hepatozoon* spp. in 29 dogs (20.4%; CI 14.6–27.8%), *Mycoplasma* spp. in 15 dogs (10.6%; CI 6.5–16.7%), *Anaplasma* spp./*Ehrlichia* spp. in 11 dogs (7.7%; CI 4.4–13.3%), and *Babesia* spp. in six dogs (4.21%; CI 1.9–8.9%). Co-infections were found in 13 dogs (9.1%; CI 5.4–15.0%) (Table 2). Sequencing performed for each positive sample revealed presence of *Babesia vogeli* (2.81%), *Babesia canis* (1.41%), *Hepatozoon canis* (20.42%), *Mycoplasma haematoparvum* (2.11%), *Mycoplasma haemocanis* (8.45%), *Anaplasma platys* (7.04%), and *Ehrlichia canis* (0.7%). DNA from *Wolbachia* spp. (MT815707), identical to Wolbachia endosymbiont of *Dirofilaria immitis* (acc. no. CP046578) amplified with the same primers used to detect *Anaplasma* spp./*Ehrlichia* spp., was detected in one dog (0.7%; CI 0.12–3.8%).

**Table 2:** Single and mixed infection of CVBD pathogens from 48 dogs (group 1: clinically suspected of vector-borne diseases) and 94 dogs (group 2: apparently healthy)

Pathogens	No. positive dogs (%)		DDBJ accessions	BLAST matching
	Group 1 (*n* = 48)	Group 2 (*n* = 94)		
***Single infections***	6 (12.5%; CI 5.9–24.7)	25 (26.6%; CI 18.7–36.3%)		
*Babesia canis*	2 (4.2%; CI 1.2–14.0)	–	MT821184	KY359360 100%
*Babesia vogeli*	1 (2.1%; CI 0.4–10.9)	3 (3.2%; CI 1.1–9.0%)	MT821127	FJ200218 100%
*Hepatozoon canis*	4 (8.3%; 3.3–19.6)	13 (13.9%; CI 8.3–22.2%)	MT821480	KP715301 99.66%
*Mycoplasma haemocanis*	1 (2.1%; CI 0.4–10.9)	6 (6.4%; CI 3.0–13.2%)	MT816510	KP715860 100%
*Anaplasma platys*	1 (2.1%; CI 0.4–10.9)	3 (3.2%; CI 1.1–9.0%)	MT815595	CP046391 100%
*Ehrlichia canis*	–	1 (1.1%; CI 0.2–5.8%)	MT815600	MN922610 100%
***Co-infections***	1 (2.1%; CI 0.4–10.9)	12 (12.8%; CI 7.5–21.0%)		
*Hepatozoon canis* + *Anaplasma platys*	1 (2.1%; CI 0.4–10.9)	4 (4.3%; CI 1.7–10.4%)		
*Hepatozoon canis* + *Mycoplasma haemocanis*	–	5 (5.3%; CI 2.3–11.8%)		
*Hepatozoon canis* + *Mycoplasma haematoparvum*	–	2 (2.1%; CI 0.6–7.4%)	MT816509	GQ129114 100%
*Anaplasma platys* + *Mycoplasma heamatoparvum*	–	1 (1.1%; CI 0.6–7.4%)		

*n*: total number

As shown in Table 2, the prevalence of *H. canis* was statistically higher compared to the other vector-borne pathogens such as *A. platys* (*P* = 0.0115), *B. vogeli* (*P* = 0.0115), and *E. canis* (*P* = 0.0013). Co-infections including *H. canis* were found in 12 dogs (8.45%). Concurrent infections of this protozoan with other canine pathogens are common, and in this study, it was strongly associated with *M. haemocanis* infections (5.32%). Nevertheless, a 4.25% prevalence of co-infections with *A. platys* and 2.13% with *M. haematoparvum* was also found.

The number of dogs that tested positive, using conventional PCR (cPCR) and stained blood smear evaluation, is represented in Table 3. Six positive samples tested positive for *Babesia* spp. using PCR; in two of them large piroplasms merozoites were also detected by light microscopy. Agreement between blood smear examination and PCR results was substantial, with approximated kappa values for *Babesia* spp. (*κ* = 0.66) and *Hepatozoon* spp. (*κ* = 0.61) and moderate kappa values for *A. platys* (*κ* = 0.43). For all these pathogens, the cPCR results presented a higher number of positives than stained blood smear microscopy.

**Table 3:** Number and percentage of dogs positive for *Babesia* spp. *Hepatozoon* spp., *Ehrlichia* spp./*Anaplasma* spp., and *Mycoplasma* spp. by microscopy of peripheral blood in both groups

Pathogen agent	Cytology		PCR	
	Clinically suspected of vector-borne diseases	Apparently healthy	Clinically suspected of vector-borne diseases	Apparently healthy
*Babesia* spp.	1/48 (2.08)	1/94 (0.01)	3/48 (6.25)	3/94 (3.19)
*Hepatozoon* spp.	5/48 (10.42)	13/94 (13.83)	5/48 (10.41)	24/94 (25.53)
*Ehrlichia* spp./*Anaplasma* spp.	1/48 (2.08)	4/94 (4.25)	5/48 (10.41)	9/94 (9.57)
*Mycoplasma* spp.	1/48 (2.08)	12/94 (14.89)	1/48 (2.08)	14/94 (14.89)

According to registration form data, none of the dogs present in this study had a history of travel to central or northern Portugal, or other countries. Data collected from each clinical case in group 1 have shown that the most common diagnostic method performed to diagnose babesiosis, ehrlichiosis, and rickettsiosis are serological tests. Antibodies against *Babesia* spp. were detected in 14 of the 25 dogs (56%) tested from group 1. None of those dogs were positive by PCR or had merozoites detected by the stained blood smear. Interestingly, one of the dogs that tested positive by PCR and blood smear was negative by indirect immunofluorescence. The agreement between indirect immunofluorescence testing and PCR for *Babesia* spp. was poor (*κ* = −0.0807). From 20 animals previously tested by IFAT for *Ehrlichia* spp., one was positive (5%) with a titer to 1:50 but had a negative result by PCR and by blood smear microscopy.

For the dog that tested positive for *Wolbachia* spp., filaria forms were observed by microscopy of the blood smear. A serological test (Witness^®^Dirofilaria, Zoetis) was performed by the clinician and confirmed the positive result for *D. immitis*.

Clinical signs in dogs from group 1 infected with different pathogens are presented in Table 4. In dogs infected with *B. canis*, *B. vogeli*. *H. canis*, *M. haemocanis,* and *A. platys*, the most common clinical signs were lethargy and anorexia. Regarding haemorrhages/coagulation abnormalities, one dog positive for *H. canis* presented with epistaxis and one dog infected with *A. platys* presented with severe ecchymosis and oedema in both hind limbs. Splenomegaly was observed by abdominal ultrasonography in one dog infected with *B. vogeli* and another dog co-infected with *H. canis* and *A. platys*. The dog infected with *D. immitis* presented clinical signs of lethargy, anorexia, fever, and proteinuria on urinalysis, including culture and sensitivity test. Due to a positive serology test for *D. immitis*, thorax X-rays, abdominal ultrasonography, and echocardiography were performed, and were unremarkable.

**Table 4:** Summary of clinical presentation in 10 dogs from southern Portugal, clinically suspected of CVBD (group 1) and with a positive result and molecular characterisation by sequence analysis

Pathogens	Clinical signs							
	Lethargy	Anorexia	Vomit	Pale mucous membranes	Hyperthermia (> 39 °C)	Haemorrhages	Splenomegaly	Musculoskeletal changes
*Babesia canis 1*	+	+	+	–	+	–	–	–
*Babesia canis 2*	+	+	+	–	–	–	–	–
*Babesia vogeli*	+	+	+	+	+	–	+	–
*Hepatozoon canis* 1	−	–	–	+	–	Epistaxis	n.d.	–
*Hepatozoon canis* 2	+	–	–	–	+	–	–	Tetraparesis
*Hepatozoon canis* 3	–	+	+	+	–	–	n.d.	–
*Hepatozoon canis* 4	+	+	–	–	–	–	n.d.	–
*Mycoplasma haemocanis*	+	+	–	–	–	–	n.d.	–
*Anaplasma platys*	+	+	+	–	+	Ecchymosis	–	–
*Hepatozoon canis *+* Anaplasma platys*	–	+	–	+	–	–	+	Weakness of the limbs

−: not found; +: present; n.d.: not determined

Significant haematologic changes in the complete blood count of infected dogs are presented in Table 5. Thrombocytopenia, confirmed by manual platelet count, was associated with single infections of *B. canis, A. platys*, *D. immitis*, and co-infection of *H. canis* with *A. platys*. Anaemia was found in four of the five dogs (80%) infected with *H. canis*. Anaemia present in dogs infected with *B. canis* and *B. vogeli* was classified as normochromic and normocytic from haematology and microscopy findings. Significant leucocytosis was associated with single infections of *H. canis*, *A. platys*, and *D. immitis*.

**Table 5:** Significant haematological changes present in 10 dogs from southern Portugal, clinically suspected of CVBD and with a positive molecular result and followed by sequence analysis characterisation

Pathogens	Haematologic changes			
	Platelets	Haematocrit	Leucocytes	
	Thrombocytopenia < 200 × 10^3^/ml	Anaemia HT < 37%	Leucocytosis > 17 × 10^3^/ml	Leucopenia < 6 x 10^3^/ml
*Babesia canis 1*	189	~	~	~
*Babesia canis 2*	18	~	~	~
*Babesia vogeli*	~	21.2	~	~
*Hepatozoon canis* 1	~	33.3	~	~
*Hepatozoon canis* 2	~	23.9	~	~
*Hepatozoon canis* 3	~	36.7	44.35	~
*Hepatozoon canis* 4	~	~	21.60	~
*Mycoplasma haemocanis*	~	~	~	~
*Anaplasma platys*	98	~	24.25	~
*Hepatozoon canis *+* Anaplasma platys*	8.64	18.1	~	~

~: in reference values

Of the 13 dogs with single infections of *H. canis* in the group of apparently healthy, five of them (38.46%) presented with hyperthermia, and four of them (30.77%) with mild anaemia. However, of the dogs with co-infections associated with *A. platys*, only two presented hyperthermia. All the dogs with co-infections had a PCV within the normal range.

## Discussion

This study includes two groups to obtain better insight on the CVBD pathogens and possible co-infections in dogs from southern Portugal. Infections of *Ehrlichia* spp., *Mycoplasma* spp., *Babesia* spp., *Hepatozoon* spp., and *Mycoplasma* spp. were detected by PCR and microscopy.

*Hepatozoon canis* was the most prevalent pathogen detected by PCR and sequencing in both groups, in agreement with other molecular studies from the north and southern Portugal, but higher than 3.1% in other studies from southern Portugal [12] and 0.9% in France [28]. A higher prevalence of *H. canis* (75.6%) was detected by PCR in red foxes (*Vulpes vulpes*) in Portugal, the presumptive reservoir of this pathogen for domestic dogs, and this shows that it is widespread in this region [29]. Some of the other countries with high molecular prevalence of dogs infected with *H. canis* include Nepal, Malta, and Cape Verde with prevalence rates of 31.43% [18, 30], 19% [31], and 35.9% [32], respectively. Co-infections of *H. canis* in this study were associated mainly with *Anaplasma platys* [30–32].

The molecular prevalence of *Anaplasma* spp./*Ehrlichia* spp. in the current work (4.25%) was higher than a previous study in southern Portugal in a similar sampled population that showed a prevalence of 1.9% [12] and similar to the 4.0% obtained in Spain [33] and the 3.7–6% in Italy [34]. So far, molecular prevalence of *Mycoplasma haemocanis* in Portugal compared to Mediterranean countries seems to be higher [18], supported by findings in this study (6.4%). To the best of our knowledge, this is the first study that detected the specie *Mycoplasma haematoparvum* in Portugal. Furthermore, this pathogen was co-infected with *A. platys* and *H. canis*. DNA of *Wolbachia* spp. was found in one dog (0.7%) from group 1, suggestive of filarial nematode infection such as *Dirofilaria immitis* and *Dirofilaria repens* [35]. Following the clinical approach, this positive result for *Wolbachia* spp. was confirmed by a serological test (Witness^®^Dirofilaria), positive for *Dirofilaria immitis*.

Although DNA detection of *Babesia canis*, *Babesia vogeli*, and “*Babesia vulpes*” has already been reported in dogs from the north of Portugal [10] and *B. vogeli* in dogs from south of the country [36], this is the first study that reports the presence of *B. canis* in southern Portugal. The high molecular prevalence of *B. canis* in northern Portugal is as expected since its vector, *Dermacentor reticulatus*, is more abundant in that area [37, 38]. Since the animals did not have a history of travel, the detection of *B. canis* in southern Portugal can be explained by import of the infected ticks, the habitat expansion of *D. reticulatus*, or its adaptation of vector transmission to *Ixodes ricinus* [37, 39]. This can also be expected by increasing spread of *D. reticulatus* and the findings of a significant *B. canis* infection rate in ticks from southern Europe [40]. The molecular prevalence of *B. vogeli* (3.19%) is higher than the 0.9% found in France [28] and 2% in Spain [33].

The high molecular prevalence of co-infections in both groups (12.7%) was expected since they are often in endemic areas [36]. A previous study [12] that collected samples from healthy animals from 2011 to 2014 in southern Portugal only detected one co-infection and a lower molecular prevalence of these pathogens using PCR and sequencing. An increase of both single and co-infections is expected nowadays due to changes in the environment, such as global climate change, urbanisation, and habitat encroachment. These changes also increase the probabilities of contact with ticks and/or sylvatic reservoir hosts. The transport of animals from non-endemic areas to endemic areas and vice versa highlights how geographic expansion can encourage the spread of these pathogen vectors and increase the numbers of co-infections in the population. Also, multiple pathogens can be present in the vector and be transmitted during a single inoculation of a dog [8]. However, considering that the samples were collected from dogs in shelters, the prevalence in this study can be higher than the general population due to a possible higher risk of exposure to the vectors in kennels. Dogs from group 2 demonstrated a higher prevalence of infections (39.4%) when compared with group 1 (14.6%) using PCR. This could be expected due group 2's living conditions in infrastructures and surrounding vegetation, higher density of the population, and poor ectoparasite control [18, 41]. Considering the limited study area, more studies are needed to identify endemic and non-endemic areas in southern Portugal.

Clinical signs in dogs infected with tick-borne pathogens can vary from mild to life-threatening and are not pathognomonic [15]. Two dogs positive for *A. platys* presented in the hospital with clinical signs of anorexia and pale mucous membranes. Interestingly, the dog with only *A. platys* infection had a severe clinical presentation of ecchymosis in both hind limbs, thrombocytopenia, and neutrophilic leucocytosis compatible with canine thrombocytic anaplasmosis [27]. These infections are commonly asymptomatic, but cases such as this show the importance of inclusion of this disease in clinical differential diagnosis in persistent thrombocytopenia. The evidence of haemolysis on serum analysis was found only in two co-infections with *A. platys* + *H. canis* in each group, which suggests this co-infection can potentiate disease pathogenesis altering clinical manifestations [1]. The negatively synergistic pathogenesis associated with co-infections of CVB pathogens has been described. It results in a complex disease expression, impairing the achievement of a definitive diagnosis and selection of proper therapeutic agents [41, 42].

Single infections of *H. canis* were found in dogs in group 1 presented in consult, with clinical signs and CBC changes compatible with hepatozoonosis [17] such as lethargy (2/4), anorexia (2/4), anaemia (3/4), and neutrophilic leucocytosis (2/4). Although no symptoms were noted by owners or kennel assistants in dogs in group 2, dogs infected with *H. canis*, 5/13 dogs were found to be hyperthermic, and 4/13 were found to have mild anaemia. Curiously, most of the co-infections with *H. canis* were found in the group of apparently healthy dogs with no changes in the physical exam and microhaematocrit (10/11). This suggests that most of these co-infections can be subclinical but still have the potential to progress to severe disease, so should not be neglected.

Pathogenicity of Mycoplasma species in dogs is still debated [18]. In the current study, dogs with a single infection of *M. haemocanis* presented with mild clinical signs of lethargy and anorexia, but no changes in CBC were noted. This is similar to dogs infected with *M. haemocanis* and *M. haematoparvum* from the apparently healthy group. In accordance with previous studies, the pathogenic potential of canine haemoplasma in the sampled dogs appeared to be low [18].

*Babesia canis* infection was detected in two dogs, both belonging to group 1, with a history of anorexia and lethargy of 2–4 days. The acute clinical manifestations were compatible with acute babesiosis including thrombocytopenia (2/2), fever (1/2), suggestive non-regenerative anaemia (1/2), and lymphopenia (2/2) [43]. In contrast, previously reported clinical findings of dogs infected by *B. vogeli* include anorexia, lethargy, fever, suggestive non-regenerative anaemia, lymphopenia, and splenomegaly [14]. In the group of apparently healthy dogs, only one presented with hyperthermia on physical exam and a microhaematocrit within the normal range. Generally, chronic carriers of *B. vogeli* do not show clinical signs unless their health deteriorates as a result of immunosuppressive treatment, splenectomy, or other immune-compromised circumstances [15].

The dog from group 1 infected with *Wolbachia* spp. was diagnosed with *Dirofilaria immitis* infection and had compatible clinical signs with dirofilariasis. Although there was no evidence of pulmonary hypertension or cardiac changes, the dog presented proteinuria on urinalysis, likely secondary to glomerulonephritis associated with microfilaraemia.

The diagnosis of *Babesia* spp. infections is often based on intraerythrocytic piroplasm observation in peripheral blood smears and serology-based diagnosis, which is very common in clinical practice in Portugal. Nevertheless, serology-based diagnosis as an indirect immunofluorescence test lacks specificity due to antigenic cross-reactivity [44] and can give false positives in endemic areas [45]. In the current study, 14 dogs (56%) were seropositive, but both PCR and blood smear observations were negative. This could be a result of the limitations of the serology-based test described or indicates previous exposure to infection. DNA of *B. canis* was detected in one dog by cPCR, which was negative by indirect immunofluorescence test, possibly due to an acute infection [15, 46]. As expected, the concordance between indirect immunofluorescence testing and cPCR was poor, given both techniques detect different factors. In contrast, the concordance between PCR and peripheral blood smear observation results was substantial for *Babesia* spp. diagnosis, belonging to the large piroplasms group.

Fifteen of the 29 dogs PCR-positive for *H. canis* were positive by microscopic observation of peripheral blood smears. In a previous study, DNA of *H. canis* was detected in 70/331 dogs from Portugal, but in only 62 dogs could gamonts be observed in buffy coat smears [13]. Since levels of parasitemia of the pathogen are intermittent and low parasitemia is often present in subclinical infections, molecular methods should be applied in routine diagnostic procedures to give a more accurate diagnosis [1]. Due to the number of co-infections found in this area, the use of multiplex PCR technique is recommended to detect simultaneously different pathogens [47].

As the optimal treatment options differ between diseases, veterinarians are required to choose CVBD diagnostic tests wisely and accurately evaluate exposure to and/or infection with a spectrum of vector-borne pathogens [15, 48].

## Conclusions

The two groups included in this study give a good representation of the pathogens and possible co-infections of dogs in Lisbon, southern Portugal. In the group with dogs suspected of CVBD from the veterinary hospital, infections of *Hepatozoon canis* and *Anaplasma platys* were detected, with compatible signs of hepatozoonosis and thrombocytic anaplasmosis. These pathogens are not usually included in differential diagnostics in clinical practice, and this study raises awareness for the need of regular molecular diagnosis to assist resident veterinarians in their clinical approach.

The high prevalence found within this study, when compared to previous studies from Portugal, can be explained by the greater exposure of animals to vectors and eminent spread of these pathogens in the population.

## Data Availability

Not applicable.

## Abbreviations

CVBD: Canine vector-borne disease
VBD: Vector-borne disease
CBC: Complete blood count
PCR: Polymerase chain reaction
EDTA: Ethylenediamine tetraacetic acid
PCV: Packed cell volume
cPCR: Conventional polymerase chain reaction
